# An *in vitro* model to assess effects of a desensitising agent on bacterial biofilm formation

**DOI:** 10.1080/23337931.2018.1544847

**Published:** 2018-12-24

**Authors:** Jamie Coulter, Nicholas S. Jakubovics, Philip M. Preshaw, Matthew J. German

**Affiliations:** Centre for Oral Health Research, School of Dental Sciences, Newcastle University, Newcastle-Upon-Tyne, England, UK.

**Keywords:** Dentine hypersensitivity, *Streptococcus mutans*, dental caries susceptibility

## Abstract

Desensitising agents are added to dentifrices to occlude exposed dentine tubules and reduce pain associated with dentine hypersensitivity. In occluding the tubules these agents may alter the surface layer of the dentine and consequently affect bacterial biofilm formation. This research sought to examine the effects of desensitising agents on dentinal biofilms using an in vitro model. A constant depth film fermenter (CDFF) was selected to mimic the oral environment and human dentine with exposed tubules was analysed. Calcium sodium phosphosilicate (CSPS) was selected as a model desensitising agent. Dentine discs were treated with pumice or CSPS-containing dentifrices with or without fluoride, or left untreated (control). Dual-species biofilms of *Streptococcus mutans* and *Streptococcus sobrinus* were grown in artificial saliva and analysed by viable counts, polymerase chain reaction (PCR) and scanning electron microscopy (SEM). SEM images confirmed the presence of occluded tubules after CSPS application and demonstrated the formation of biofilms containing extracellular matrix material. Analysis of PCR and viable count data using a one-way ANOVA showed no significant differences for bacterial composition for any of the four treatments. There were, however, trends towards increased numbers of bacteria for the pumice and CSPS treated samples which was reversed by the addition of fluoride to CSPS. In conclusion, CSPS was not found to have a significant effect on biofilms and an *in vitro* model for testing desensitising agents has been developed, however, further work is required to improve the reproducibility of the biofilms formed and to explore the trends seen.

## Background

Dentine hypersensitivity occurs when a tooth with exposed dentine interprets relatively innocuous stimuli as noxious [1]. One explanation for dentine hypersensitivity is the hydrodynamic theory; this stipulates that a stimulus causes movement of fluid within dentine tubules resulting in nerve depolarisation and a painful stimulus. Thus, dentine tubules exposed to temperature changes or air pressure could result in fluid movement and pain [2]. Some treatments such as the desensitising agents added to dentifrices focus upon blocking or occluding the dentinal tubules in an attempt to reduce sensitivity. One product which works via this mechanism is calcium sodium phosphosilicate (CSPS). This forms a layer of carbonated hydroxyapatite crystals on dentine when in contact with an aqueous environment [3]. CSPS occludes significantly more dentine tubules and decreases dentine permeability significantly more than other control toothpastes [4,5].

Although desensitising agents occlude tubules they also form a layer on the surface of dentine. This, therefore, means there is the potential to alter the entire tooth surface through the deposition of a layer on top of dentine after treatment with CSPS, which has been shown *in vitro* to be between 3 and 5 mm thick [6]. *In vivo* this layer may be thinner, but any alternation to the surface may affect the salivary pellicle which forms on dentine made up from the selective adhesion of salivary biopolymers such as glycoproteins [7]. This in turn could affect the adhesion of bacteria to the pellicle and the subsequent co-adhesion of other bacteria to the initial microorganisms, leading to polymicrobial biofilm formation [8].

One previous paper has explored the effect of desensitising agents on bacterial biofilms and found desensitising agents increased bacterial adhesion compared to no treatment, but this did not compare against a non-desensitising dentifrice [9]. Our study seeks to explore the difference between desensitising and non-desensitising containing dentifrices, hypothesising that any changes to the surface layer of a tooth may impact upon the microbial biofilm which forms and possibly affect the tooth’s caries risk. To assess this we developed an *in vitro* method to examine the effect of desensitising agent CSPS on exposed dentine using dual-species biofilms containing *S. mutans* and *S. sobrinus*.

## Methods

### Sample preparation

Molar and premolar human teeth samples were obtained from a local tissue bank stored at Newcastle Dental Hospital following the regulations of the UK Human Tissue Act 2004 with consent being obtained from donors and samples stored in a safe, secure and anonymous fashion [10]. Dentine discs were prepared using a 5 mm internal diameter diamond drill piece (Eternal Tools, Oxfordshire, UK), then divided in two using an annular diamond blade (Microslice 2, Metal Research, Cambs, UK) and lapped using a PM2A lapping machine (Logitech, Glasgow, UK) with 3 µm Calcium Aluminium Oxide powder (Logitech). To expose dentinal tubules, samples were immersed in citric acid formulated at 6% *w/v* for 90 sec. Samples were polished for 15 sec with light pressure using a slow-speed hand piece (NSK, Tokyo, Japan) and a rubber polishing cup (Dentsply, Pennsylvania, USA). Samples were then randomly allocated to four groups, namely they were left untreated as a control or polished with either pumice, a CSPS containing dentifrice or a CSPS containing dentifrice with fluoride. The operator was blinded for fluoride but could not be blinded for CSPS content due to differences in colour.

### SEM analysis

A selection of samples were analysed using scanning electron microscopy (SEM). These were fixed in 2% gluteraldehyde in Sorensons Phosphate Buffer (TAAB Lab. Equipment, Aldermaston, UK) then rinsed in Sorensons Phosphate Buffer and dehydrated. Samples were dried using a critical point drier (Baltec, Leica Microsystems, Milton Keynes, UK), mounted onto sticky carbon discs with Achesons Silver Dag (Agar Scientific, Essex, UK) and sputter coated with gold of 15 nm thickness using a Polaron SEM Coating Unit. Samples were examined using a Stereoscan 240 electron microscope (Cambridge Instruments, Cambridge, UK).

### Saliva preparation

Parafilm stimulated saliva was collected from five healthy volunteers. Dithiothreitol (DTT) was added and saliva was pasteurised and processed as per methods from other studies [11]. Artificial saliva was based upon a previous recipe [12]. The solution was autoclaved following which 1 mL of 40% 0.2 µm filter sterilized urea (Sigma-Aldrich, St Louis, USA) solution was added.

### Bacterial inoculum

To standardise the inoculum of *S. mutans* and *S. sobrinus*, 2 × 100 ml of THYE broth were made using (per L) 36.4 g Todd Hewitt Broth (Bacto, New South Wales, Australia), 5 g Yeast Extract (Bacto) and 1 L of distilled water. Broths were inoculated with *S. mutans* UA159 [13] and 100 mL THYE with *S. sobrinus* NCTC 12279. These were cultured to mid-exponential phase (OD_600nm_ = 0.5), harvested by centrifugation at 3800 g and 4 °C for 10 min and re-suspended in artificial saliva. Cells were stored in 1 mL aliquots at -20 °C. Cell concentrations were estimated by viable counting to ensure concentration of 5 × 10^8^ CFU/ml.

### Growing the biofilms

Discs were recessed into pans in a constant depth film fermenter (CDFF) [11] by 300 µm and the entire CDFF sterilised by autoclaving. The CDFF was placed within an incubator set to 30 °C with ambient air without shaking and 10 mL of saliva was injected into the CDFF. The CDFF was left running for 1 h to allow a salivary pellicle to form, after which a 2 mL standardised innoculum containing 1 mL of *S. mutans* inoculum and 1 mL of *S. sobrinus* inoculum was injected into the CDFF. This was followed by 15 mL of 2% sucrose solution. A reservoir of artificial saliva was attached along with a peristaltic pump running at a speed of 0.5 mL per minute (resting salivary flow rate) allowing artificial saliva to drip over the samples for 24 h. A solution of 2% (*w/v*) sucrose was administered 4 times in 24 h to mimic the sugar that would be present in the mouth from a naturally occurring diet [14]. After 24 h, the samples were removed aseptically, placed in 1 mL of sterile PBS and vortexed vigorously for 1 min to remove attached bacteria.

### Viable counts

Following vortexing, viable counts were performed by serially diluting the samples 10-fold in sterile PBS, and each dilution was spot inoculated onto THYE Agar plates 3 times. Plates were incubated at 37 °C for 48 hours after which colony forming units were counted using the differences in colony morphology to distinguish between *S. mutans* and *S. sobrinus*.

### Extraction of DNA for PCR analysis

Following removal of cells from discs, samples were centrifuged at 13,800 g and 4 °C for 10 min and the supernatant removed before adding 150 µl of spheroblasting buffer, comprising 20 mM Tris–HCl, pH 6.8, 10 mM MgCl_2_, 26% (*w/v*) raffinose.5H_2_O. Mutanolysin (3.33 µg/mL) and lysozyme (16.7 µg/mL) were added. The solution was incubated at 37 °C for 30 minutes before adding 150 µL 2× T&C lysis solution (Epicentre MasterPure™ DNA Purification Kit, Illumina, Wisconsin, USA). Samples were transferred to a screw cap Eppendorf containing 50 mg of 1.5 mm acid washed glass beads, placed in a TissueLyser LT (QIAGEN, Hilden, Germany) and shaken at 50 Hz for 5 min. DNA was extracted using the Epicentre MasterPure™ DNA Purification Kit in accordance with the manufacturer’s instructions.

### Quantitative PCR (qPCR)

Each qPCR reaction well contained 3 µl of the prepared DNA sample, 7.5 µl of 2× SensiMix™ SYBR No-ROX MasterMix (Bioline, London, UK), 1.875 µl Forward Primer (2 µM),1.5 µl Reverse Primer (2 µM) and 1.2 µl Distilled H_2_O. Primers MKD F/R (gene *gtfD*) were used for *S. mutans* and SobGTFI F/R (gene *gtfI*) were used for *S. sobrinus* (Table 1). Each extracted DNA sample was run in three reaction wells on each qPCR plate. Standard curves containing template DNA of either *S. mutans* or *S. sobrinus* were included in each run, along with negative control wells containing all reagents except DNA template.

**Table 1.** Forward and reverse sequences of the primers MKD and SobGTFI.

Primer	Forward sequence	Reverse sequence
*MKD*	GGCACCACAACATTGGGAAGCTCAGTT	GGAATGGCCGCTAAGTCAACAGGAT
*SobGTFI*	GATAACTACCTGACAGCTGACT	AAGCTGCCTTAAGGTAATCACT

The qPCR was performed using a DNA Engine 2 Opticon Continuous Fluorescence Detector (MJ Research, Quebec, Canada) and a plate reading was taken after each cycle. The results were analysed using the Opticon 2.0 program (MJ Research). The protocol used was 95 °C for 10 min to activate polymerase followed by 39 cycles of 95 °C for 15 sec to denature; 58 °C for 30 sec to anneal; 72 °C for 1 min 30 sec for elongation. This was optimised to consistently give a primer efficiency of approximately 86% for SobGTFI and 80% for MKD. The qPCR data were converted into the amount of genome copies present to provide a measure of the amount of bacteria on each disc. Molar concentrations of standards were calculated by quantifying DNA standards using a ND-1000 Spectrophotometer (ThermoFisher Scientific) and converting into number of molecules using the molecular weight of the *S. mutans* and *S. sobrinus* genomes.

## Results

### Preparation of dentine discs with exposed tubules

**Figure 1.**
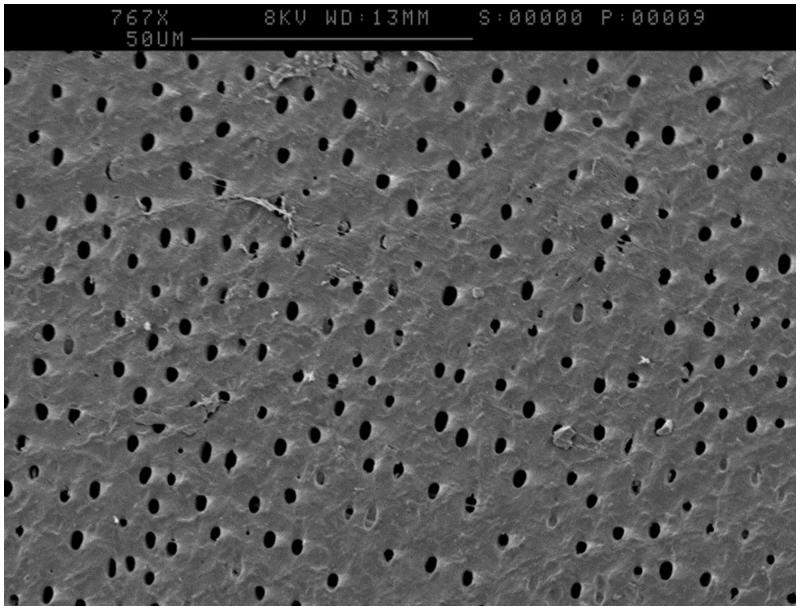
Patent dentine tubules following etching of the dentine surface with 6% citric acid for 90 seconds.

Imaging via SEM highlighted that the lapping process had created a smear layer thought to be caused by aluminium oxide, data not shown. Etching with 6% citric acid for 90 seconds removed this layer and revealed dentine tubules as shown in Figure 1.

### Treatment of prepared discs

**Figure 2.**
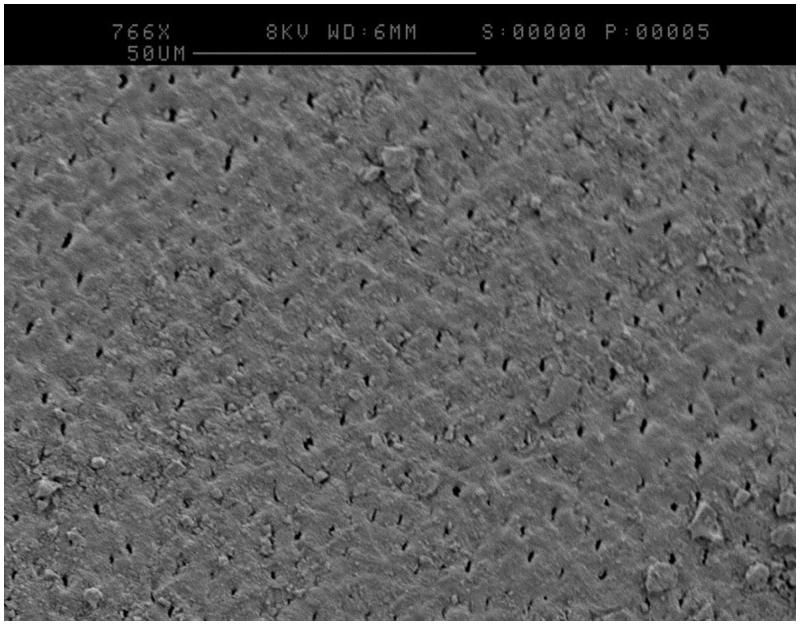
SEM image of a CSPS plus fluoride treated sample following incubation showing occluded tubules and an altered surface layer.

**Figure 3.**
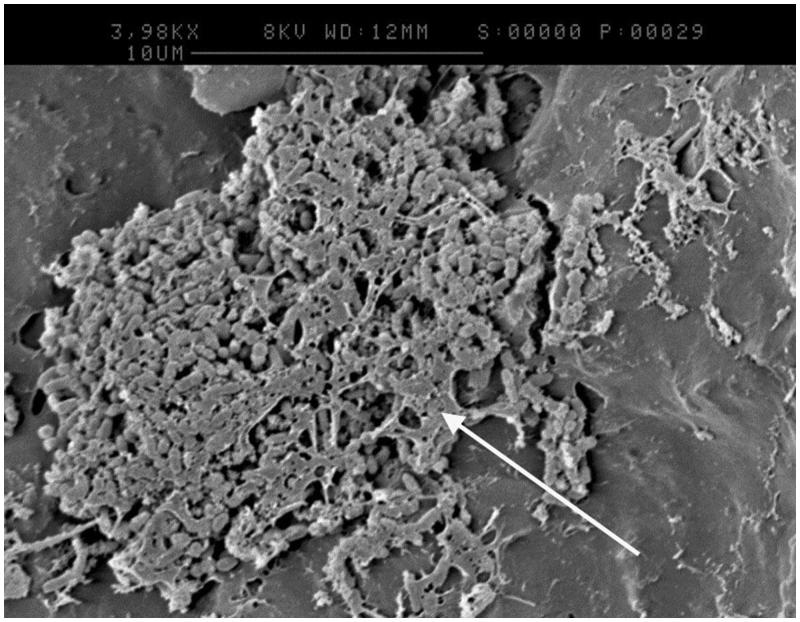
SEM image of a pumice treated sample following intubation demonstrating the formation of an extracellular matrix (highlighted by arrow).

One disc from each treatment was analysed by SEM after incubation in the CDFF for 24 h under flowing artificial saliva. Figure 2 was taken of a CSPS plus fluoride treated sample at high magnification and appeared to show tubule occlusion. Figure 3 was taken of a pumice treated sample and appeared to show the formation of an extracellular matrix (white arrow), which has collapsed down onto the surface of the bacteria due to the dehydration process.

### Effects of densensitising agent on biofilm formation

**Figure 4.**
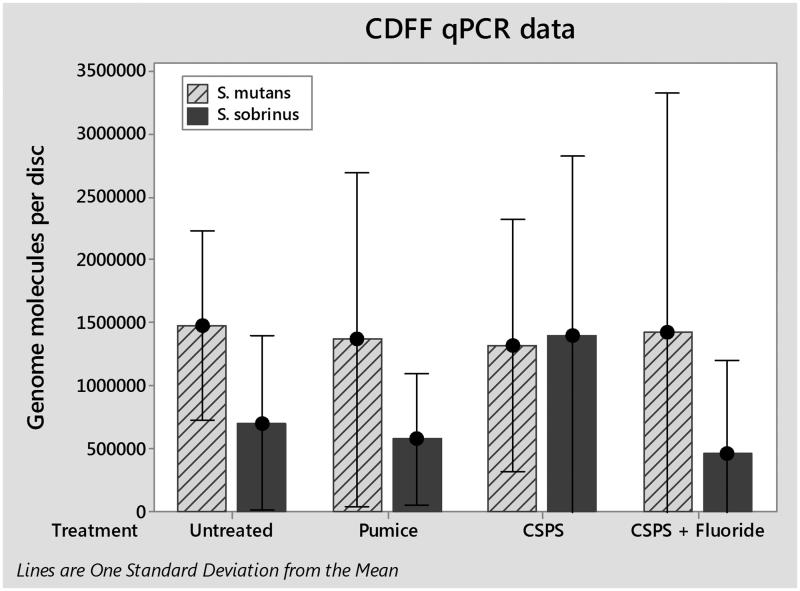
qPCR estimates for the number of *S. mutans* and *S. sobrinus* bacteria per a sample. Error Bars represent one standard deviation from the mean.

**Figure 5.**
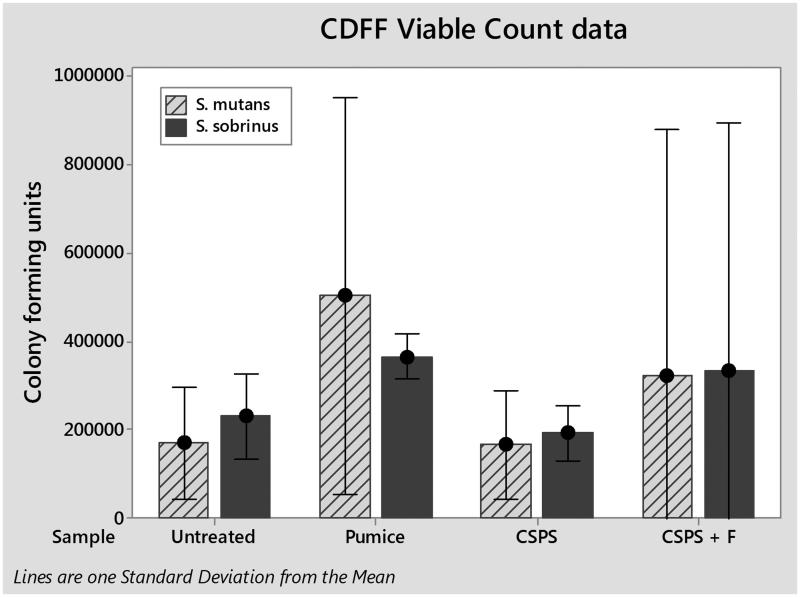
Colony forming units estimated on each sample as the number per a disc for* S. mutans* and *S. sobrinus*. Error bars represent one standard deviation from the mean.

The total numbers of *S. mutans* and *S. sobrinus* cells in each biofilm were quantified by qPCR (Figure 4). One way analysis of variance (ANOVA) found no significant difference between either species for any treatment; *S. mutans* (F(3, 8) = 0.01, *p*>.05) and *S. sobrinus* (F(3,8) = 0.64, *p*>.05). Viable cells in biofilms were determined by viable counts (Figure 5). Again, one-way ANOVA found no significant differences for any treatment; *S. mutans* (F(3, 8) = 0.56, *p*>.05) and *S. sobrinus* (F(3,8) = 0.24, *p*>.05).

## Discussion

A CDFF was selected to model cariogenic biofilms due to its similarities to the oral environment. Specifically, biofilms are grown on a solid surface and nutrients are provided in a thin film which is constantly replenished [15]. CDFFs have been used previously in dental research, for example to test the demineralisation of dentine [11] or how the roughness of denture material effects biofilm formation [16]. However, these studies have used bovine tooth tissue, synthetic hydroxyapatite or restorative materials which do not represent a dentinal surface with exposed tubules that would receive CSPS treatment. Creating dentine discs from human teeth has resolved this issue, and patent dentine tubules were clearly observed. CSPS-containing dentifrices were selected as an example of a desensitising agent and SEM images such as Figure 2 revealed tubule occlusion and deposition of a surface layer following its use, something which appears to remain during biofilm growth. These images agree with what has occurred in previous CSPS *in vivo* research [4] and so it appears that the model mimics the action of desensitising agents in the clinical environment.

Mutans streptococci, *S. mutans* and *S. sobrinus* were used as a simplified cariogenic biofilm as they are commonly associated with dental caries [17]. Thought to be linked to their acidophilic nature and their ability to interact and form biofilms by synthesising extracellular polysaccharides (EPS) including insoluble glucans from sucrose [18]. Insoluble glucans affect caries risk as they provide reserve fermentation material for acid production, increase biofilm thickness, trap acid near the tooth and aid the adherence of bacteria to biofilms [19]. Extracellular matrix material was observed in Figure 3, and this may represent EPS such as insoluble glucans since biofilms were cultured in the presence of sucrose.

Viable counts and qPCR were employed on biofilms produced to provide estimates of the viable and total numbers of cells present, respectively. Overall, there were relatively low numbers of cells in biofilms on the discs, which is consistent with the SEM observations which showed patchy coverage across the surfaces. In the untreated (control) samples, there was a moderate correlation between qPCR and viable counts, although, in general, counts were higher when measured by qPCR than by viable counts. It is likely due to there being some non-viable cells present that were not detected by viable counts. This is shown by i*n vivo* studies using confocal laser scanning microscopes demonstrating that for some biofilms 15% of bacteria were vital at the biofilm base compared to about 70% of bacteria at the top [20].

Initial analysis of the data obtained indicates that there were no significant differences in biofilm composition between any of the treatments for either viable counts or qPCR when compared using a one-way ANOVA. One issue may be that the low numbers of cells present and lack of confluent coverage led to a high degree of variability in the data. Future studies will aim to reduce this variability producing thicker and more robust biofilms, by growing the biofilms over 72 h instead of 24 h to ensure confluence under SEM. This will then be tested against established methods from the literature such as *in vivo* mounted samples which whilst close to the clinical scenario are more complex to run due to cost and ethical considerations.

Despite these future changes to the methodology there were trends seen within the data such as for viable counts of *S. mutans* and *S. sobrinus* on pumice-treated samples to be nearly double those on untreated surfaces. Whilst this is not significant it may represent an important difference which could be explored with greater numbers in future research. This may be caused by an increase in roughness since there is evidence that pumice tends to roughen the surface of dentine [21] and it has been shown that increasing roughness above a threshold increases the size of the biofilms formed [22].

Trends within qPCR results show a large increase in *S. sobrinus* on the CSPS-treated samples compared to the untreated average. This may be due to increases in surface free energy and roughness in the presence of a CSPS layer as increases in both parameters with dental implants caused an increase in biofilm formation [23]. Any increase in *S. sobrinus* on CSPS-treated dentine appeared to be reversed by the addition of fluoride. This may be caused by the well-known bacteriostatic effects of fluoride [24], although previous studies employing the CDFF model found that pulsing *S. mutans* biofilms with 135 ppm fluoride twice a day had no effect on biofilm vitality [14]. It is possible that *S. sobrinus* is more sensitive to fluoride than *S. mutans* under the conditions employed in our model as the fluoride is present on the surface layer.

One previous paper has explored the effect of desensitising agents on bacterial biofilms and found that desensitising agents increased bacterial adhesion when applied with an electric toothbrush compared with no treatment, application by hand or fluoride varnish [9]. Our study differs from this result and did not find any significant change in bacterial adhesion. It should be noted that there were important differences in the design of the two studies. Cury et al. [9] used bovine dentine and did not culture biofilms under fluid flow, which is important for replenishing nutrients and removing waste. In addition, saliva was not used and the study did not feature a positive control for tooth brushing. Our model using the CDFF integrated fluid flow, used human dentine, employed natural human saliva to form a pellicle, and contained controls of pumice and untreated samples. Whilst there is clearly scope to modify and improve our model, we believe it has many advantages and has demonstrated that it can be used to quantify vital and total cells within dual-species cariogenic biofilms. In developing and refining this model we will be able to examine various other desensitising agents in future tests and explore further the trends seen within this study.

## Conclusion

Statistical analysis of the qPCR and viable count data found none of the treatments to be significantly different from each other, indicating that CSPS pastes cause no significant change to biofilm formation compared to control dentifrices. However, on examining the data trends were found towards increased numbers of *S. mutans* and *S. sobrinus* in some circumstances that are worthy of further exploration with larger sample numbers. Importantly, an *in vitro* model for testing the impact of desensitising agents on dentine biofilms has been developed using the CDFF to mimic the oral environment, dentine discs with exposed dentine tubules and Mutans streptococci to represent the cariogenic challenge. Future experiments will aim to improve the reproducibility of the biofilms and will compare the effects of different desensitising agents on oral biofilm formation in the CDFF and against other established models of biofilm formation such as those using samples mounted on intra-oral devices.
